# *Borrelia* infection and risk of celiac disease

**DOI:** 10.1186/s12916-017-0926-1

**Published:** 2017-09-15

**Authors:** Armin Alaedini, Benjamin Lebwohl, Gary P. Wormser, Peter H. Green, Jonas F. Ludvigsson

**Affiliations:** 1 Department of Medicine, Columbia University Medical Center, New York, NY USA; 2 Celiac Disease Center, Columbia University Medical Center, New York, NY USA; 3 Institute of Human Nutrition, Columbia University Medical Center, New York, NY USA; 4 Department of Medical Epidemiology and Biostatistics, Karolinska Institutet, Stockholm, Sweden; 5 Division of Infectious Diseases, Department of Medicine, New York Medical College, Valhalla, NY USA; 6 Department of Pediatrics, Örebro University Hospital, Örebro, Sweden; 7 Division of Epidemiology and Public Health, School of Medicine, University of Nottingham, Nottingham, UK

**Keywords:** Celiac disease, Lyme disease, Infection, Inflammation, *Borrelia burgdorferi*

## Abstract

**Background:**

Environmental factors, including infectious agents, are speculated to play a role in the rising prevalence and the geographic distribution of celiac disease, an autoimmune disorder. In the USA and Sweden where the regional variation in the frequency of celiac disease has been studied, a similarity with the geographic distribution of Lyme disease, an emerging multisystemic infection caused by *Borrelia burgdorferi* spirochetes, has been found, thus raising the possibility of a link. We aimed to determine if infection with *Borrelia* contributes to an increased risk of celiac disease.

**Methods:**

Biopsy reports from all of Sweden’s pathology departments were used to identify 15,769 individuals with celiac disease. Through linkage to the nationwide Patient Register, we compared the rate of earlier occurrence of Lyme disease in the patients with celiac disease to that in 78,331 matched controls. To further assess the temporal relationship between *Borrelia* infection and celiac disease, we also examined the risk of subsequent Lyme disease in patients with a diagnosis of celiac disease.

**Results:**

Twenty-five individuals (0.16%) with celiac disease had a prior diagnosis of Lyme disease, whereas 79 (0.5%) had a subsequent diagnosis of Lyme disease. A modest association between Lyme disease and celiac disease was seen both before (odds ratio, 1.61; 95% confidence interval (CI), 1.06–2.47) and after the diagnosis of celiac disease (hazard ratio, 1.82; 95% CI, 1.40–2.35), with the risk of disease being highest in the first year of follow-up.

**Conclusions:**

Only a minor fraction of the celiac disease patient population had a prior diagnosis of Lyme disease. The similar association between Lyme disease and celiac disease both before and after the diagnosis of celiac disease is strongly suggestive of surveillance bias as a likely contributor. Taken together, the data indicate that *Borrelia* infection is not a substantive risk factor in the development of celiac disease.

## Background

Celiac disease is an autoimmune enteropathy with genetic, environmental, and immunologic components [1]. It is characterized by an aberrant immune response to ingested wheat gluten and related proteins of rye and barley that leads to inflammation and tissue damage in the small intestine [2]. Celiac disease is strongly associated with genes for the specific class II human leukocyte antigens (HLAs) DQ2 and DQ8 [3]. It is estimated to affect close to 1% of the American population [4, 5], compared with up to 2–3% in Sweden and Finland [6, 7]. Because it can occur at any age, factors other than genes and gluten ingestion are believed to play a significant role in disease onset. In addition, recent studies point to an increasing prevalence of celiac disease in the USA and Europe within the past few decades [8–10], underscoring the potential contribution of environmental factors. Among these, exposure to infectious agents, particularly those affecting the gastrointestinal tract, and the risk of celiac disease have been examined in several studies [11–14], but the results are not consistent, and a firm conclusion has not been reached [15, 16]. A recent study demonstrates that the inflammation caused by reovirus infection can contribute to the development of autoimmunity in celiac disease through the suppression of peripheral regulatory T cell conversion [17].

Lyme disease, also known as Lyme borreliosis, is caused by the *Borrelia burgdorferi* sensu lato spirochetal bacteria. *B. burgdorferi* sensu stricto is the primary cause of Lyme borreliosis in the USA, whereas *B. afzelii*, *B. garinii*, and *B. burgdorferi* are the causative agents in Europe [18]. Lyme disease is currently the most common vector-borne infection in the USA and Europe, where its incidence has been rapidly increasing in recent years [19, 20]. Although antibiotic therapy resolves clinical symptoms in the majority of cases, a fraction of patients will develop persistent joint inflammation that does not respond to antibiotic treatment [21]. The antibiotic-refractory Lyme arthritis is believed to be the result of *Borrelia* infection-induced autoimmunity and is associated with autoantibodies [22, 23], an acute-phase inflammatory response [24], and the class II major histocompatibility complex (MHC) molecule HLA-DR4 [25]. The condition often responds to immunomodulatory or anti-inflammatory agents [26].

The potential for the contribution of infection with *Borrelia* to a later onset of celiac disease or gluten sensitivity has recently garnered attention and is discussed in various Internet forums [27–29]. In addition, recent studies from the USA and Sweden examining the regional variation in the frequency of celiac disease have demonstrated some similarity with the geographic distribution of Lyme disease. In the USA, celiac disease has been found to be most prevalent in the Northeast and the Midwest regions [30], where the great majority of cases of *B. burgdorferi* infection also occur [19]. In Sweden, the highest incidence of celiac disease has been associated with the southern region [31], where Lyme disease is endemic [32]. Despite the similar pattern of geographic distribution and the publicized conjecture regarding a potential link between celiac disease and Lyme borreliosis, studies to assess such an association are lacking. In this study, we use a population-based approach to examine whether infection with *Borrelia* increases the risk of celiac disease.

## Methods

### Study population

For this study, celiac disease was defined as a patient’s having villous atrophy (Marsh 3 histopathology grade) [33]. Earlier validation found that 96–100% of all gastroenterologists and pediatricians in Sweden performed a biopsy before celiac disease diagnosis during the study period and that in cases of reported villous atrophy, a comorbidity other than celiac disease was rare [34]. Data on villous atrophy were obtained from computerized biopsy reports from all of Sweden’s 28 pathology departments. Biopsies had been carried out in 1969–2008, while the data collection took place in 2006–2008. Biopsy data included personal identity number, date of biopsy, topography (duodenum and jejunum), and morphology. The biopsy reports were on average based on three tissue specimens [35], which would be expected to detect 95% of all celiac disease cases [36]. Biopsy was required for celiac disease diagnosis throughout the study period. Biopsy data were linked through the unique personal identity number [37] to inpatient and hospital-based outpatient data with respect to a previous diagnosis of Lyme disease.

Exposure to *Borrelia* infection was defined as having the relevant International Classification of Diseases (ICD) code for Lyme disease in the Swedish Patient Register (ICD-10: A69.2) [38]. The registry began in 1964 and became nationwide in 1987. The Patient Register was limited to inpatient data until 2000, but since 2001 it has also contained hospital-based outpatient data. The ICD code for Lyme disease was introduced in 1997.

We matched each individual with celiac disease with up to five controls for sex, age, county, and calendar year. Controls were selected by the Statistics Sweden government agency, using the Swedish Total Population Register [39].

Since information regarding diagnosis of Lyme disease was available starting on January 1, 1997, we only included study participants on or after January 1, 1998 (i.e., so that all participants had the potential to be exposed to a Lyme disease diagnosis for at least one year). This resulted in 15,769 individuals with celiac disease and 78,331 matched controls.

### Statistical analysis

We used conditional logistic regression to calculate odds ratios (ORs) for later celiac disease among individuals with Lyme disease. The conditional approach means that each individual with celiac disease was only compared with his/her matched control (within a stratum). In this way, age, sex, county, and calendar year do not affect risk estimates. Furthermore, we examined the risk of celiac disease according to the time since the diagnosis of Lyme disease (<1, 1–4.99, and ≥ 5 years). We also adjusted for country of birth (Nordic vs. not Nordic) and educational level using four a priori-defined categories [40]. Four percent of study participants lacked data on educational level and were fitted into a separate fifth category in the multivariate analysis.

To assess the temporal relationship between celiac disease and Lyme disease, we also examined the risk of later Lyme disease in patients with an initial diagnosis of celiac disease. This analysis was restricted to 15,742 individuals without a prior Lyme disease diagnosis at first biopsy with celiac disease and 78,230 matched controls. Cox regression was used to estimate the hazard ratio (HR). The follow-up period continued for each celiac disease patient and control until the first Lyme disease diagnosis, death, emigration, or December 31, 2009.

We used SPSS 22 (SPSS, Inc.) for all analyses. The OR and HR findings with 95% confidence intervals that did not overlap with 1.0 were regarded as statistically significant.

### Ethics

This study was approved by the Ethics Review Board of Stockholm, Sweden. According to the board’s decision, no study participant was contacted, and the study was strictly register-based [41].

## Results

The demographic characteristics of the study participants are shown in Table 1. The median year of study entry was 2002, and the median age at celiac disease diagnosis (and corresponding age in matched controls) was 32 years (range 0–95).

**Table 1:** Characteristics of patients with celiac disease and matched controls

Characteristic	Main analysis^a^		Prospective analysis^b^	
	Matched controls	Celiac disease	Matched controls	Celiac disease
No. of participants	78,331	15,769	78,230^b^	15,742^b^
Sex				
Male, no. (%)	28,934 (36.9)	5837 (37.0)	28,890 (36.9)	5826 (37.0)
Female, no. (%)	49,397 (63.1)	9932 (63.0)	49,340 (63.1)	9916 (63.0)
Age^c^				
≤ 19 years, no. (%)	29,510 (37.7)	5917 (37.5)	29,455 (37.7)	5900 (37.5)
20–39 years, no. (%)	15,746 (20.1)	3167 (20.1)	15,738 (20.1)	3167 (20.1)
40–59 years, no. (%)	17,245 (22.0)	3464 (22.0)	17,226 (22.0)	3460 (22.0)
≥ 60 years, no. (%)	15,830 (20.2)	3221 (20.4)	15,811 (20.2)	3215 (20.4)
Calendar period^c^				
1998–2002, no. (%)	39,492 (50.4)	7956 (50.5)	39,476 (50.5)	7952 (50.5)
2003–2008, no. (%)	38,839 (49.6)	7813 (49.5)	38,754 (49.5)	7790 (49.5)
Lyme disease, no. (%)	73 (0.09)	25 (0.16)	215 (0.27)	79 (0.50)

^a^To assess the risk of celiac disease following a diagnosis of Lyme disease

^b^To assess the risk of later Lyme disease in patients with a diagnosis of celiac disease. We excluded patients with an earlier diagnosis of Lyme disease. In addition, two celiac disease patients with potential data irregularity were excluded

^c^At the time of celiac disease diagnosis

Twenty-five (0.16%) individuals with celiac disease and 73 (0.09%) controls had a record of earlier Lyme disease (OR, 1.61; 95% CI, 1.06–2.47) (Table 1). Adjustment for the level of education and Nordic origin did not substantially alter the risk (adjusted OR, 1.58; 95% CI, 1.03–2.41). The OR was highest in the first year after Lyme disease diagnosis (OR, 1.87; 95% CI, 0.87–4.00) but remained positive between 1 and 5 years (OR, 1.48; 95% CI, 0.84–2.60) and thereafter (OR, 1.72; 95% CI, 0.51–5.81). ORs for celiac disease were similar in men (OR, 1.64; 95% CI, 0.81–3.32) and women (OR, 1.60; 95% CI, 0.94–2.72) (*P* for interaction, 0.962).

In the prospective analysis, we found that 79 individuals with celiac disease (0.50%) and 215 controls (0.27%) had a record of later Lyme disease (HR, 1.82; 95% CI, 1.40–2.35) (Table 1). Adjustment for the level of education and Nordic origin had no substantial effect (adjusted HR, 1.82; 95% CI, 1.40–2.37). The risk of Lyme disease was highest in the first year of follow-up (HR, 2.18; 95% CI, 1.07–4.44) but remained significantly increased between 1 and 5 years after celiac disease diagnosis (HR, 1.97; 95% CI, 1.41–2.77), and positive thereafter (HR, 1.44; 95% CI, 0.88–2.34). HRs for Lyme disease were not significantly different in men (HR, 2.39; 95% CI, 1.71–3.56) and women (HR, 1.51; 95% CI, 1.08–2.133) (*P* for interaction, 0.134). The relative risk in the prospective analysis was not significantly different from that in the main analysis.

## Discussion

The strengths of this study are its nationwide population-based design and high statistical power. The prevalence of celiac disease in Sweden is among the highest in the world [6], making the setting of this study particularly relevant. Furthermore, the majority of the Swedish population lives in areas where Lyme disease is endemic [32]. Because Lyme borreliosis is not a notifiable infection in Europe, accurate estimates of its current incidence in Sweden are not available. However, a recent study found a mean annual incidence of 464 cases of erythema migrans (a skin lesion indicative of the early stage of Lyme disease) per 100,000 inhabitants in southeastern Sweden between 1997 and 2002, with a sharp increase in the rate being observed during the analyzed period [20]. In neighboring Norway, a recent study of anti-*B. burgdorferi* IgG seroprevalence (indicative of possible ongoing or past episode of Lyme disease) reported a rate of about 4% [42].

Infectious agents have been linked to the pathogenesis of a number of autoimmune diseases, with one or more mechanisms, such as molecular mimicry, epitope spreading, bystander activation, and suppression of regulatory T cell function, being implicated [43, 44]. In this study, we found a modest association between celiac disease and a prior record of Lyme borreliosis. However, we also found celiac disease to be similarly associated with a subsequent occurrence of the infection. Furthermore, both associations were strongest in the first year after diagnosis. As such, the observed modest association between celiac disease and Lyme disease is almost certainly due to surveillance bias, driven by the possibility that physicians would be more likely to investigate a patient presenting with *Borrelia* infection for markers of celiac disease (and vice versa). Given that there is some overlap between the systemic symptoms of Lyme disease and celiac disease, it is reasonable to assume that such surveillance bias would have increased the probability of celiac disease diagnosis in this study.

We acknowledge that patients with celiac disease may be different from controls in a number of ways that have not been measured. Though our cases were matched to controls by age, sex, county, and calendar year, there could be differences between cases and controls in relationship to both celiac disease and Lyme borreliosis which may contribute to the observed results. Therefore, while strongly suggestive of surveillance bias as the likely source for the association, other components, such as shared genetic, environmental, or other factors between celiac disease and Lyme disease might be potential additional contributors. For example, a variable previously found to be associated with Lyme borreliosis and celiac disease is income [45, 46]. Furthermore, it is important to note that the results and conclusions of this study cannot be extrapolated to forms of gluten sensitivity other than celiac disease.

## Conclusions

In summary, we found that, despite the high rates of occurrence of both celiac disease and Lyme disease in the population for this study, only a minor fraction of the celiac disease patients had an earlier record of Lyme disease. Furthermore, the modest relationship between Lyme disease and celiac disease both before and after the diagnosis of celiac disease points to surveillance bias or shared risk factors as the likely contributors. Taken together, our data argue against a causal association of *Borrelia* infection with celiac disease.

## Abbreviations

*B. afzelii*: Borrelia afzelii
*B. burgdorferi*: Borrelia burgdorferi
*B. garinii*: Borrelia garinii
CI: confidence interval
HLA: human leukocyte antigen
HR: hazard ratio
ICD: International Classification of Diseases
MHC: major histocompatibility complex
OR: odds ratio
